# Artificial Intelligence Versus Traditional Learning in a Medical School Setting

**DOI:** 10.7759/cureus.85262

**Published:** 2025-06-02

**Authors:** Anya Ramsamooj, Steven M Ibrahim, Valerie A Gerriets, John K Cusick, Rajendra Ramsamooj

**Affiliations:** 1 Pediatrics, California Northstate University College of Medicine, Elk Grove, USA; 2 Anesthesiology, California Northstate University College of Medicine, Elk Grove, USA; 3 Pharmacology, California Northstate University College of Medicine, Elk Grove, USA; 4 Basic Sciences, California Northstate University College of Medicine, Elk Grove, USA; 5 Pathology, California Northstate University College of Medicine, Elk Grove, USA

**Keywords:** artificial intelligence, flipped classroom, medical education, traditional teaching, voice-over

## Abstract

Introduction: As technology advances in every aspect of our lives, educational curricula are moving away from traditional didactic lectures toward more interactive modalities. While there are many ways to incorporate non-traditional learning methods into medical curricula, the increasing prevalence of Artificial Intelligence (AI) offers a novel avenue. This study aims to determine whether the use of AI enhances learning as compared to more traditional flipped classroom styles, such as lecture slides and voice-over videos.

Methods: Incoming first-year medical students were given learning materials prior to course instruction. Participating students were randomly assigned to three educational groups: PowerPoint slides only (PPT), voice-over videos and PowerPoint slides (VO+PPT), or Artificial Intelligence learning and PowerPoint slides (AI+PPT). After reviewing the materials, all study participants took a 22-question formative quiz. The average overall scores and the scores on the five most “difficult” questions were assessed between groups.

Results: Sixty-two incoming students participated in this study. There was no significant change between groups regarding overall scores. The PPT group had significantly lower scores on the five most “difficult” questions when compared to the VO+PPT (p = 0.026) and AI+PPT (p = 0.004) groups. However, there were no significant changes in scores on the five most “difficult” questions between the VO+PPT and AI+PPT groups (p = 0.834).

Conclusions: The VO+PPT and AI+PPT groups performed similarly but significantly better than the PPT-only group regarding performance on “difficult” questions. Overall, our study provides evidence that Artificial Intelligence and voice-over modalities are equally viable methods of learning, depending on the demands of professors and students.

## Introduction

In recent years, medical school curricula have been moving away from traditional didactic lectures to more active learning modalities. One commonly used modality includes the “flipped classroom,” in which students are presented with lecture material before in-class instruction, permitting more time in class to focus on interactive activities, such as review games, group discussions, and Socratic exchange with the professor [1]. Previous work has shown that flipped classrooms were favored over traditional classrooms among students and showed greater success in learning performance [2]. Due to the broad spectrum of teaching methods, there are many ways to integrate the “flipped classroom” style of learning. In medical education, quizzes, review games, and case-based learning are all used in the flipped classroom format to foster active learning [3].

In one example, Stanford University School of Medicine used pre-recorded ethics lectures prior to class. Rather than conducting a traditional lecture, in-class time was more interactive and provided direct physician-patient interaction regarding the pre-recorded topics. The study found that students felt more confident and had a deeper understanding after the activity [4]. However, it is difficult to apply these findings to the entirety of medical education, as it was an ethics-based topic rather than a scientific or clinical concept. Another study examined student learning in physiology courses and found that flipped classrooms utilizing videos and online resources prior to in-class time enhanced learning compared to traditional lectures [5]. Nevertheless, the degree of benefit is difficult to pinpoint and may vary due to variations in learning modality preferences of both faculty and students.

While there are many different ways to incorporate a flipped classroom learning style into medical curricula, the increasing prevalence of Artificial Intelligence (AI) offers another avenue [6]. Several recent studies suggest that both educators and students believe that AI learning will eventually become the main modality of learning [7,8], as many educators and students believe that it is the most effective way to learn in areas of medicine, bioengineering, and other fields [9].

AI is often presented as having somewhat human attributes, which may enhance learning compared to simply searching for a question online [10]. For students who may not be comfortable directly interacting with professors, it may also offer them a comfortable, non-confrontational space to gain the necessary knowledge. Another potential benefit is that AI can present information in multiple forms to enhance the learning style of the student. However, few studies have sought to determine if and how to best incorporate AI into the curriculum. Thus, this study examines whether AI is beneficial to students in their first exposure to biomedical topics, as it is used in “flipped learning” or used by students to ask for deeper insights and connect the concepts to solidify the content. Overall, this study aims to determine if the use of AI enhances learning compared to more traditional flipped classroom styles, such as lecture slides and voice-over videos.

## Materials and methods

Study population

This study was approved by the Institutional Review Board (IRB) at California Northstate University College of Medicine (CNUCOM) (IRB protocol number: 2012-02-77). Inclusion criteria encompass all incoming first-year medical students for the Class of 2027 at California Northstate University College of Medicine (CNUCOM).

Study design

All incoming students in the Class of 2027 were invited via email to participate in the present study. After confirming their interest in participation, students were required to sign a consent form administered via SurveyMonkey (http://www.surveymonkey.com). All incoming students were given introductory learning material prior to the start of their first course, Foundations of Clinical Medicine. The pre-matriculation content included Cell Biology, Immunology, Biochemistry, Pharmacology, Microbiology, Histology, Pathology, and Medical Terminology. Faculty used a range of techniques to create the voice-over lectures, including Camtasia, Microsoft PowerPoint, and Panopto software.

**Figure 1:**
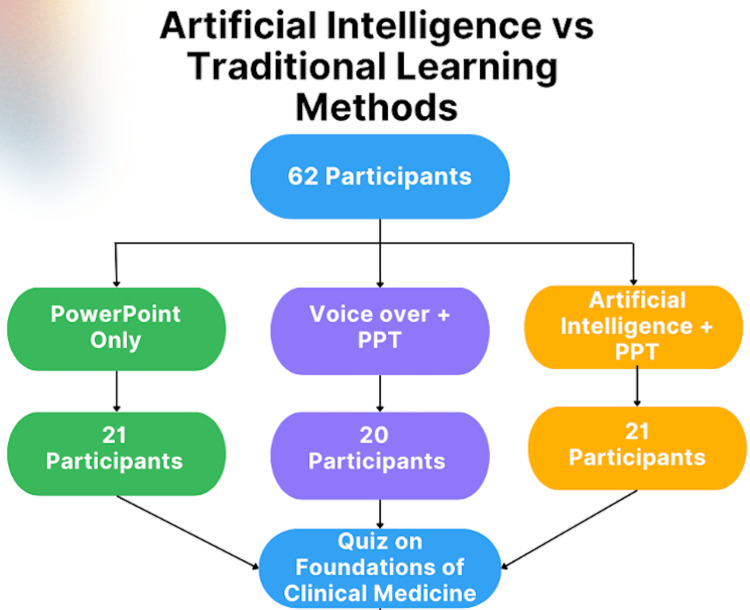
Flowchart depicting the study design

As detailed in Figure 1, only the 62 participating students were randomly assigned into three educational groups: PowerPoint slide only (PPT), voice-over videos and PowerPoint slides (VO+PPT), or Artificial Intelligence learning and PowerPoint slides (AI+PPT). The AI+PPT group was composed of the PPT with narration and real-time interaction with edYOU (Los Angeles, CA). Twenty-one students were in the PPT group, 20 students were in the VP+PPT group, and 21 students were in the AI+PPT group.

After reviewing the pre-matriculation materials, all study participants were required to answer a 22-question formative quiz to assess their knowledge. This pre-matriculation quiz was developed by Dr. John K. Cusick, Dr. Valerie A. Gerriets, and Dr. Rajendra Ramsamooj and can be found in the Appendices. The primary endpoint measured in the present study was performance on a formative quiz. Students were given a modest gift card worth $25 as an incentive for participating in this study.

Artificial Intelligence platform

The AI learning experience was delivered by proprietary technologies designed to be personal, ethical, and educationally effective (edYOU; Los Angeles, CA). Using a Personalized Ingestion Engine (PIE), the platform curates diverse learning materials from expert sources worldwide. A Personalized AI (PAI) then leverages natural language processing to tailor customized conversations to each student’s current knowledge. In addition, an Intelligent Curation Engine ensures that the AI interacts safely using techniques such as content flagging, toxicity blocking, and data verification. This combination enables AI tutors on the platform to build long-term mentoring relationships by adapting to each learner’s evolving needs. The version of edYOU utilized in this study was provided with the learning goals of the pre-matriculation content specified by the faculty members who created the content; the learning goals of this study were specific to the pre-matriculation content of CNUCOM for the Class of 2027.

Quiz score analysis

The average overall scores on the formative quiz were assessed for each group. Additionally, the average scores on the five most “difficult” questions were assessed for each group. The most “difficult” questions were defined as the five questions that had the lowest percent correct.

Statistical analysis

An ordinary one-way analysis of variance (ANOVA) test was performed to compare the effectiveness of the different learning modalities by comparing the overall average scores of each group on the formative quiz, as well as the five most difficult questions. Tukey’s multiple comparison test was then used to further compare the different groups. Type I error is set to 0.05.

A power analysis was conducted for a balanced one-way ANOVA with three groups to detect a large effect size (effect size = 0.4) with a significance level of 0.05 and a desired power of 0.8. This indicated that the sample size should be 66, with 22 students per group. However, because only 62 students agreed to participate in the present study, 21 students were in the PPT group, 20 students were in the VP+PPT group, and 21 students were in the AI+PPT group. Therefore, the actual power of this study is less than the desired power of 0.80.

## Results

Quiz scores

**Figure 2:**
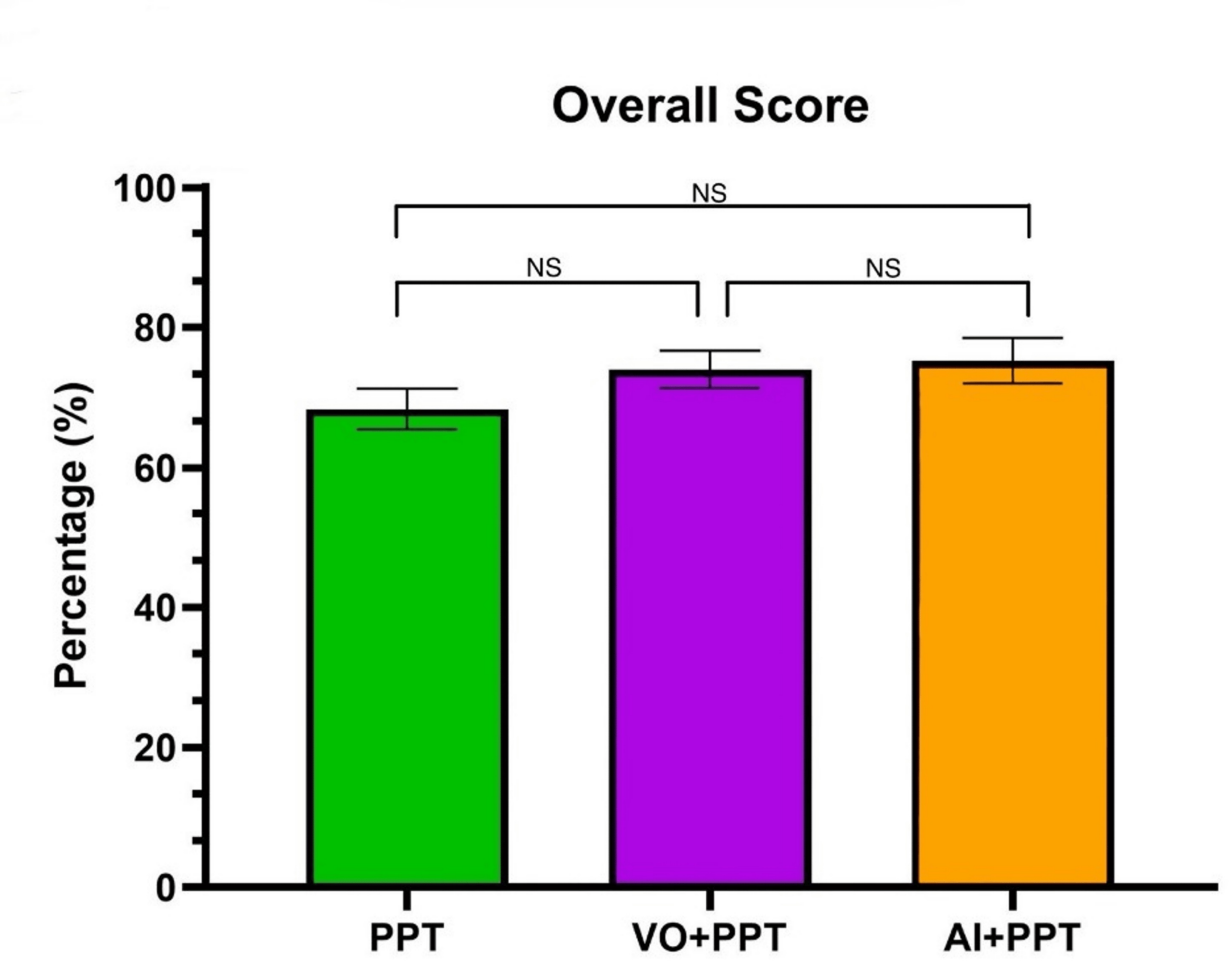
Average overall score (%) on the formative quiz for each group No significant differences were found between any of the groups (p = 0.225, using the one-way ANOVA test). A p-value < 0.05 is statistically significant. PPT: PowerPoint-only group, VO+PPT: voice-over + PowerPoint group, AI+PPT: Artificial Intelligence + PPT, NS: not significant, ANOVA: analysis of variance

On the overall formative quiz, students in the PowerPoint-only group (PPT) scored 68.3 ± 2.9%, students in the voice-over and PowerPoint (VO+PPT) scored 74.0 ± 2.7%, and students in the Artificial Intelligence and PowerPoint (AI+PPT) scored 75.2 ± 3.2% (Figure 2). The ordinary one-way ANOVA test showed no significant differences (p = 0.225).

**Figure 3:**
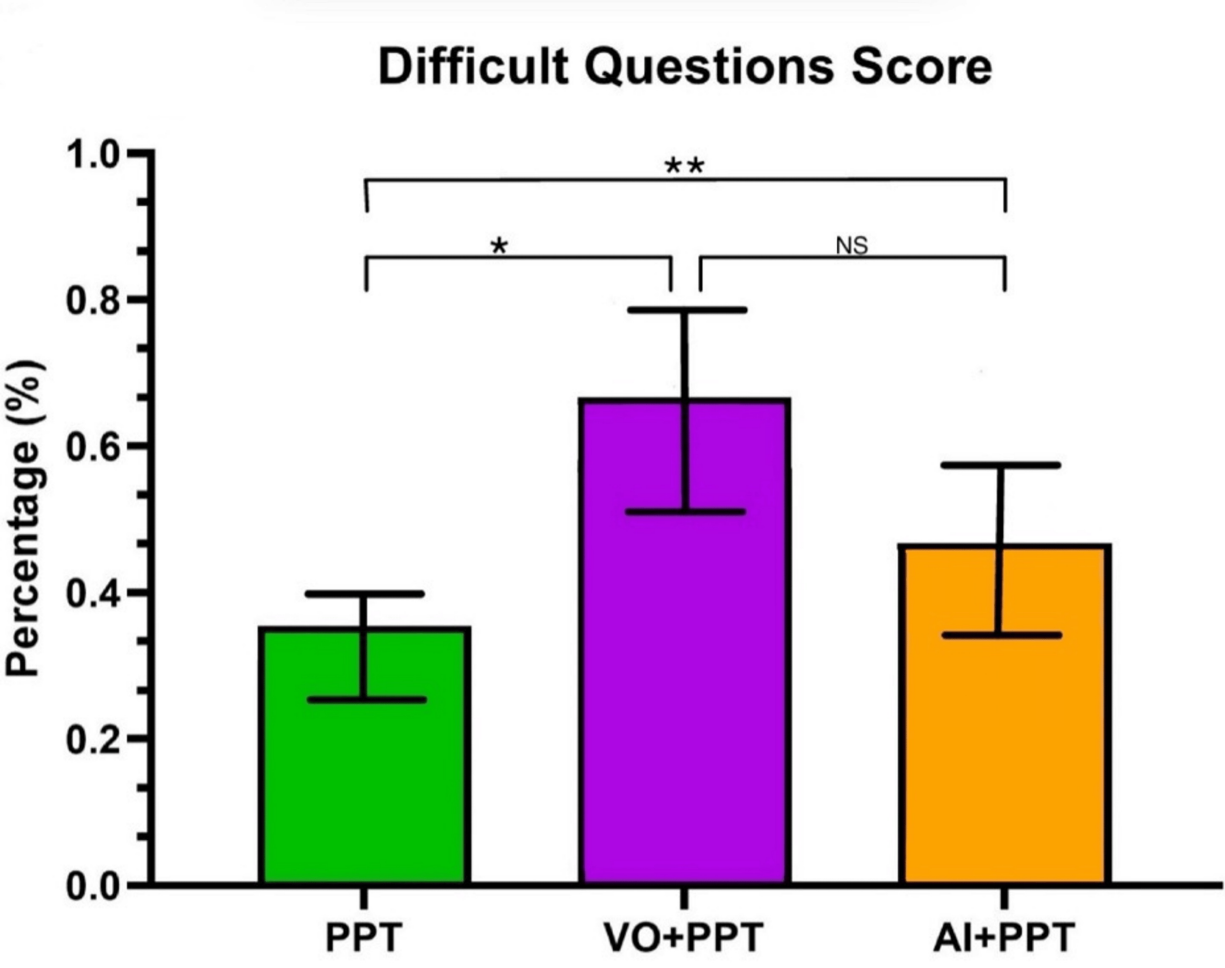
Average score (%) on the five most difficult questions on the formative quiz for each group Both the VO+PPT and AI+PPT groups scored significantly higher than the PPT-only group. A p-value < 0.05 is statistically significant. A Tukey's multiple comparison test provided a p-value of 0.026 when comparing the VO+PPT group with the PPT-only group. A Tukey's multiple comparison test provided a p-value of 0.004 when comparing the AI+PPT group with the PPT-only group. There were no significant differences between the VO+PPT and AI+PPT groups (p = 0.834 via Tukey’s multiple comparison test). PPT: PowerPoint-only group, VO+PPT: voice-over + PowerPoint group, AI+PPT: Artificial Intelligence + PPT, NS: not significant *p < 0.05, **p < 0.005

On the most “difficult” questions, students in the PPT group scored 34.9 ± 4.6%, students in the VO+PPT group scored 66.0 ± 13.8%, and students in the AI+PPT group scored 45.0 ± 12.7% (Figure 3). The ordinary one-way ANOVA test showed significant differences among means (p = 0.003). Therefore, Tukey’s multiple comparison test was performed to compare the three groups. The PPT group was significantly different from the other two groups (PPT versus VO+PPT, p = 0.026; PPT versus AI+PPT, p = 0.004). However, the difference between VO+PPT and AI+PPT was not significant (p = 0.834).

Table 1 presents the mean overall quiz scores for each group. 

**Table 1:** Mean overall quiz scores (%) for each group, with SD, group sizes, ANOVA F-statistic, and p-values Differences were analyzed using one-way ANOVA. A p-value < 0.05 is statistically significant. One-way ANOVA comparing the PPT, VO+PTT, and AI+PPT groups provided a p-value of 0.225, which is statistically insignificant. PPT: PowerPoint-only group, VO+PPT: voice-over + PowerPoint group, AI+PPT: Artificial Intelligence + PPT, SD: standard deviations, ANOVA: analysis of variance

Group	Mean score (%)	SD	Number	Test statistic	One-way ANOVA
PPT	68.3	2.9	21	F = 1.54	p = 0.225
VO+PPT	74.0	2.7	20	-	-
AI+PPT	75.2	3.2	21	-	-

Table 2 presents the mean scores of each group on the five most difficult quiz questions. 

**Table 2:** Mean scores on the five most difficult quiz questions for each group, including SD, group sizes, ANOVA F-statistic, and p-values A p-value < 0.05 is statistically significant. A one-way ANOVA found a significant difference between the PPT, VO+PPT, and AI+PPT groups, and provided a p-value of 0.003. Post hoc comparisons were made using Tukey’s HSD test. A Tukey’s multiple comparison test found a significant difference between the PPT and VO+PPT groups and provided a p-value of 0.026. A Tukey’s multiple comparison test found a significant difference between the PPT and AI+PPT groups and provided a p-value of 0.004. PPT: PowerPoint-only group, VO+PPT: voice-over + PowerPoint group, AI+PPT: Artificial Intelligence + PPT, SD: standard deviations, ANOVA: analysis of variance, HSD: honestly significant difference *p < 0.05, **p < 0.005

Group	Mean score (%) on top 5 difficult questions	SD	Number	Test statistic	One-way ANOVA	Tukey’s multiple comparison test
PPT	34.9	4.6	21	F = 6.87	p = 0.003**	-
VO+PPT	66.0	13.8	20	-	-	p = 0.026*
AI+PPT	45.0	12.7	21	-	-	p = 0.004**

## Discussion

Artificial Intelligence (AI) offers a new, advanced teaching modality that is readily accessible. This learning platform gives students some autonomy with regard to their study habits and time management. The use of AI as a potential resource is growing among students; however, recent studies question whether AI is as efficient as other modalities of flipped classrooms [11].

Our study showed that students learning from PowerPoint slides only (PPT) did not perform as well as those learning from voice-over videos and PowerPoint slides (VO+PPT) and the combined use of Artificial Intelligence learning and PowerPoint slides (AI+PPT). Despite the potential benefits that AI may offer to medical education and curricula, the present study did not find that Artificial Intelligence learning coupled with PowerPoint slides (AI+PPT) significantly enhanced overall quiz performance compared to more a traditional flipped classroom modality using voice-over PowerPoints (Figure 2), as there was no significant difference between overall scores (p = 0.225). This suggests that the students were able to gain a basic understanding of the introductory material regardless of the different teaching and learning modalities. One potential confounding factor could have been that the content presented may have some overlap with content learned before entering medical school (e.g., from undergraduate education, the Medical College Admission Test (MCAT) and not necessarily represent knowledge gained specifically from the learning modalities examined in this study). However, because all the students in the present study were required to complete the same prerequisites for admission into CNUCOM, this is unlikely to have a large impact on our findings. Another potential confounding factor is the fact that students have unique study habits and differing motivations when completing schoolwork. While this is true for many education-related studies, randomization of participants helps to minimize any effects of this.

Similar to how the overall scores on the quiz are key to understanding the advantages and disadvantages of different learning modalities, the five most difficult questions also give insight into possible topics students do not recognize and show which students not only understand the topic but have possibly mastered it. However, the present study found that the AI+PPT group performed significantly better on the five most difficult questions compared to the PPT-only group, but that this improvement was not significantly different compared to the VO+PPT group. The VO+PPT group also performed significantly better compared to the PPT-only group on the most difficult questions (Figure 3). The AI interface that was used in the present study allowed students to ask AI questions and receive immediate answers without having to turn their attention away from the learning module, which was thought to aid their overall learning. While additional research is needed to examine the role of this type of AI usage in medical education, our study suggests that students utilizing AI perform as well as students learning from voice-over PowerPoint presentations designed by faculty members.

These similarities may be due to several reasons. One potential reason why the AI group did not perform significantly better than the other groups could be a lack of familiarity with AI and technology issues. Lack of experience using AI platforms can create a learning curve, as prior knowledge of how to best utilize AI modalities and this program specifically may have enhanced its effectiveness in learning. This does not apply to other learning modalities, as the students have been attending lectures, similar to voice-overs, and using PowerPoint slides to learn since they started their educational journeys. Having to troubleshoot any technology-related problems can add stress to the student and take away from the student’s ability to learn the material. As such, if this AI modality were to be implemented into a medical school curriculum, our study suggests that it would be beneficial to both students and educators to receive training on how to best utilize the AI technology [12].

Additionally, students in the study may have found methods to gain information on their own. Although the AI+PPT group was able to ask the AI questions about the material as if there was an educator in the room with them, students in the PPT-only and VO+PPT groups may have other ways to find answers to their questions, such as simply searching for the answers online. This allows students to fill the gaps in knowledge without depending on the learning modality that they received.

Lastly, another potential explanation for the similar results may have been due to the similar nature of the AI+PPT and VO+PPT groups. Both the AI and voice-overs were similar in structure and content. In this study, the AI was only trained on the content in the voice-over PowerPoint presentations, and therefore, the AI was not able to give the extra background information, possible analogies, or practice questions that AI would usually excel at doing. Due to the nature of the study, these parameters may have limited AI to such an extent that it performed similarly to the voice-overs, therefore producing similar results. One of the more beneficial aspects of AI use in education is its adaptive learning ability, where it creates an understanding of what specific students might prefer while learning [13].

Overall, while this study did not show that AI was superior to faculty-designed voice-over PowerPoint presentations, this study confirms that AI+PPT and VO+PPT performed similarly. With this finding in mind, the AI learning modality might be beneficial as a learning modality, as it may give professors additional time in their schedules compared to designing and recording a voice-over lecture. AI integration into education curricula may allow professors to have more office hours and time in class with students, or focus on the smaller details of the lesson. Another advantage to AI integration is that information and analyses are provided instantaneously, which can increase productivity for both students and educators [14]. AI models allow for students to gain personalized learning by allowing students to have more control over their learning environment [15].

Limitations

While the results of this study suggest a potential benefit of AI learning in medical education, there were also limitations. One limitation of this study is the small sample size, as there were only 62 participants and only nine students completed the qualitative survey assessing AI. Additionally, this study lacked a qualitative survey assessing students’ views on voice-over PowerPoints for comparison. The small numbers may lack the specificity needed to make a more definitive conclusion. Additionally, this study did not examine the baseline knowledge of participants before the study, nor did not correlate student GPA or performance on standardized examinations. Another limitation of this study is that we only examined the immediate effectiveness of the AI learning modality, as the quiz was administered shortly after review of the pre-matriculation materials. Examining the long-term effectiveness of AI learning modalities is critical to examine. Previous work has detailed the value of spaced repetition in enhancing knowledge retention [16]. AI platforms that incorporate principles of spaced repetition, such as those described by Koenig et al., may further optimize retention and clinical readiness by leveraging evidence-based memory consolidation strategies [16].

Future directions

Further iterations of this study will focus on training students on how to best use AI in the context of medical learning so that the benefits of AI can be maximized. Additionally, a pre-intervention quiz can be implemented and compared to the post-intervention quiz scores to measure improvement across the groups and the benefit that can be attributed to each intervention. The long-term implications of using AI as a primary learning modality can also be explored by examining the final course grades and course examination performance within each group. The AI modality used here, edYOU, can also be compared with other AI modalities, such as ChatGPT, to determine its effectiveness in any learning outcomes, as well as student performance.

## Conclusions

This study found that supplementing traditional PowerPoint-based learning with either Artificial Intelligence (AI) or voice-over presentations significantly improved performance on the most difficult quiz questions, although no significant difference was observed in overall quiz scores among the three groups. These findings suggest that while AI is not superior to existing voice-over methods, it is equally effective and holds potential as a complementary tool to traditional medical education. Its real-time responsiveness and customizable interaction provide a flexible learning experience for students, especially those seeking personalized learning or alternative ways to engage with material. With proper training and integration into curricula, AI could reduce faculty burden and enhance the student learning process. Future iterations of this specific AI model (edYOU) should aim to overcome technical barriers and explore adaptive features that leverage AI’s unique capabilities to further support diverse learning needs.

## Appendices

The pre-matriculation quiz used in the present study is shown in Table 3. 

**Table 3:** Pre-matriculation quiz on Foundations of Clinical Medicine Credits: Dr. John K. Cusick, Dr. Valerie A. Gerriets, and Dr. Rajendra Ramsamooj

Question number	Question	Answer choices
1	Please enter the following information: (last name, first name, CNSU email address)	Free response
2	Diseases inherited in an autosomal dominant pattern feature:	a. Affected individuals in each generation, b. Equal chance of disease expression for either males or females, c. Transmission of the mutated gene from carrier mothers to ½ of their sons, d. A and B, e. None of the above
3	Exon sequences in a gene:	a. Regulate when and in which cells RNA is transcribed for that gene, b. Are “spliced” out of the final RNA sequence during production of mRNA, c. A and B, d. None of the above
4	Histologically, signs of malignancy include all of the following, except:	a. Low mitotic rate, b. Hypocellularity, c.Hyperchromatic nuclei, d. High nuclear-to-cytoplasmic ratio (N:C), e. Anisonucleosis and pleomorphism
5	When considering anisonucleosis and pleomorphism, which of the following is the most correct?	a. Terms are analogous, b. Pleomorphism describes dark nuclei, which implies malignancy, c. Anisonucleosis can be part of anaplasia, d. Are associated with benign processes, e. Synonymous with anaplasia
6	Which of the following terms specifically refers to the actions of a drug on the body?	a. Pharmacokinetics, b. Pharmacogenomics, c. Pharmacodynamics, d. Pharmacology, e. Placebo effect
7	Which of the following terms specifically refers to the actions of the body on a drug?	a. Pharmacology, b. Pharmacodynamics, c. Pharmacokinetics, d. Pharmacogenomics, e. Placebo effect
8	A woman is visiting the United States with her three‐week‐old infant and brings her in for medical care, as the baby is lethargic and presenting with diarrhea and vomiting. The mother reported that she is exclusively nursing. A physical examination shows jaundice and an enlarged liver. After switching from breast milk to infant formula containing sucrose as the sole carbohydrate, the baby’s symptoms resolve within three days. Which of the following is the most likely diagnosis?	a. Galactosemia, b. Intolerance to dietary fructose, c. Inborn error of fructose metabolism, d. Deficiency of an enzyme in glucose metabolism, e. Deficiency of an enzyme in pentose sugar metabolism
9	A 28‐year‐old woman brought her three‐day‐old baby to the hospital after giving birth at home. The infant appeared healthy at birth, but after 24 hours, the baby appeared to be in distress. Vitals showed a heart rate of 157 bpm and a respiratory rate of 81 breaths per minute. Laboratory work showed hyperammonemia, and the physician began to suspect an inborn error of metabolism. Which of the following would be the recommended treatment given the diagnosis?	a. Low-fat diet, b. High-protein diet, c. Low-carb diet, d. Low-protein diet, e. High-fat diet
10	The sequence of amino acids that makes up a protein molecule is called the:	a. Primary structure, b. Secondary structure, c. Tertiary structure, d. Quaternary structure
11	Which of the following best describes the arrangement of amino acid side chains in an alpha helix?	a. Point inward toward the center, b. Point toward the C-terminal, c. Point outward from the helical axis, d. Point toward the N-terminal
12	When cells respond to an extracellular signal, they most often convert the information from one form to another. This process is called:	a. Signal transformation, b. Signal amplification, c. Signal transduction, d. Signal interference
13	The polarity in a DNA strand is indicated by referring to one end as the 3’ end and the other as the 5’ end. Which structure is on the 3’ end and defines the 3' end?	a. Phosphate group, b. Acetyl group. c. Hydroxyl group, d. Nitrogenous base, e. Ribose sugar
14	Which of the following best describes a primary lymphoid organ?	a. Where lymphocytes undergo initial differentiation, b. Where circulating leukocytes meet, c. Where adaptive immune responses start, d. Filtration device, e. Collection of PRRs that bind antigens
15	A cell from the lymphocytic lineage undergoes early stages of differentiation within the bone marrow. Before leaving there, it begins to display IgM on its surface. The cell is most likely destined to become a:	a. Monocyte, b. Basophil, c. T helper cell, d. Neutrophil, e. B cell
16	Which of the following statements regarding HYPO-PITUITAR-ISM is true?	a. Word has a prefix, body, and suffix, b. “Hypo” from Greek “hipo,” meaning under, c. “Ism” from Greek “-ismos,” meaning condition of, d. All of the above
17	Pericarditis refers to which of the following:	a. Greek rap group from Mykonos, b. Inflammation around the heart, c. Tumor of the heart muscle, d. Infection of heart valves
18	Regarding the medical record, which of the following is FALSE?	a. Inaccuracies can make a physician look silly in court, b. Copies must be provided with patient authorization, c. Should be completed ASAP after interaction, d. Changes are allowed only in a different color ink
19	When evaluating a patient for the first time, which of the following is true?	a. Mention your tee-time, b. Dress very casually, c. Stand by the door, d. Use “buddy” or “hon”, e. Ask how they prefer to be addressed
20	With respect to lab studies, which of the following is false?	a. Pretest probability helps interpret test result validity, b. Specificity = correctly identifying those without disease, c. Any lab value outside normal = abnormality, d. Sensitivity = identifying all with the disease
21	Which of the following statements is true regarding labs?	a. Ranges differ by sex and age, b. Don’t annoy lab techs with stool requests, c. Mayo Clinic offers 120 lab tests, d. Serum = plasma
22	Bacteria that have a thick outer layer of peptidoglycan and stain purple are called:	a. Non-infectious, b. Gram positive, c. Infectious, d. Gram negative
23	Leeuwenhoek’s microscope could magnify the object up to:	a. 3000-60000 times, b. 5-10 times, c. 20-40 times, d. 50-300 times

## References

[1] Hurtubise L, Hall E, Sheridan L, Han H (2015). The flipped classroom in medical education: engaging students to build competency. J Med Educ Curric Dev.

[2] Hew KF, Lo CK (2018). Flipped classroom improves student learning in health professions education: a meta-analysis. BMC Med Educ.

[3] Nichat A, Gajbe U, Bankar NJ, Singh BR, Badge AK (2023). Flipped classrooms in medical education: improving learning outcomes and engaging students in critical thinking skills. Cureus.

[4] Periyakoil VS, Basaviah P (2013). The flipped classroom paradigm for teaching palliative care skills. Virtual Mentor.

[5] Ji M, Luo Z, Feng D, Xiang Y, Xu J (2022). Short- and long-term influences of flipped classroom teaching in physiology course on medical students’ learning effectiveness. Front Public Health.

[6] Prahani BK, Rizki IA, Jatmiko B, Suprapto N, Amelia T (2022). Artificial intelligence in education research during the last ten years: a review and bibliometric study. Int J Emerg Technol Learn.

[7] Seo K, Tang J, Roll I, Fels S, Yoon D (2021). The impact of artificial intelligence on learner-instructor interaction in online learning. Int J Educ Technol High Educ.

[8] Khayat M, Hafezi F, Asgari P, Talebzadeh Shoushtari M (2021). Comparison of the effectiveness of flipped classroom and traditional teaching method on the components of self-determination and class perception among University students. J Adv Med Educ Prof.

[9] Pupic N, Ghaffari-Zadeh A, Hu R, Singla R, Darras K, Karwowska A, Forster BB (2023). An evidence-based approach to artificial intelligence education for medical students: a systematic review. PLOS Digit Health.

[10] Pelau C, Dabija DC, Ene I (2021). What makes an AI device human-like? The role of interaction quality, empathy and perceived psychological anthropomorphic characteristics in the acceptance of artificial intelligence in the service industry. Comput Human Behav.

[11] Zawacki-Richter O, Marín VI, Bond M, Gouverneur F (2019). Systematic review of research on artificial intelligence applications in higher education - where are the educators?. Int J Educ Technol High Educ.

[12] Grassini S (2023). Shaping the future of education: exploring the potential and consequences of AI and ChatGPT in educational settings. Educ Sci.

[13] Khan RA, Jawaid M, Khan AR, Sajjad M (2023). ChatGPT - reshaping medical education and clinical management. Pak J Med Sci.

[14] Karabacak M, Ozkara BB, Margetis K, Wintermark M, Bisdas S (2023). The advent of generative language models in medical education. JMIR Med Educ.

[15] Sallam M (2023). ChatGPT utility in healthcare education, research, and practice: systematic review on the promising perspectives and valid concerns. Healthcare (Basel).

[16] Koenig ZA, Henderson JT, Brooke SM (2022). Creating a spaced repetition model to supplement education in plastic surgery. Plast Reconstr Surg Glob Open.

